# A Review on the Role of miR-1290 in Cell Proliferation, Apoptosis and Invasion

**DOI:** 10.3389/fmolb.2021.763338

**Published:** 2021-12-24

**Authors:** Soudeh Ghafouri-Fard, Tayyebeh Khoshbakht, Bashdar Mahmud Hussen, Mohammad Taheri, Mohammad Samadian

**Affiliations:** ^1^ Department of Medical Genetics, School of Medicine, Shahid Beheshti University of Medical Sciences, Tehran, Iran; ^2^ Men’s Health and Reproductive Health Research Center, Shahid Beheshti University of Medical Sciences, Tehran, Iran; ^3^ Department of Pharmacognosy, College of Pharmacy, Hawler Medical University, Kurdistan Region, Iraq; ^4^ Urology and Nephrology Research Center, Shahid Beheshti University of Medical Sciences, Tehran, Iran; ^5^ Institute of Human Genetics, Jena University Hospital, Jena, Germany; ^6^ Skull Base Research Center, Loghman Hakim Hospital, Shahid Beheshti University of Medical Sciences, Tehran, Iran

**Keywords:** miR-1290, cancer, biomarker, miRNA, expression

## Abstract

MicroRNAs (miRNAs) have been shown to affect expression of several genes contributing in important biological processes. miR-1290 a member of this family with crucial roles in the carcinogenesis. This miRNA is transcribed from *MIR1290* gene on chromosome 1p36.13. This miRNA has interactions with a number of mRNA coding genes as well as non-coding RNAs SOCS4, GSK3, BCL2, CCNG2, KIF13B, INPP4B, hMSH2, KIF13B, NKD1, FOXA1, IGFBP3, CCAT1, FOXA1, NAT1, SMEK1, SCAI, ZNF667-AS1, ABLIM1, Circ_0000629 and CDC73. miR-1290 can also regulate activity of JAK/STAT3, PI3K/AKT, Wnt/β-catenin and NF-κB molecular pathways. Most evidence indicates the oncogenic roles of miR-1290, yet controversial evidence also exists. In the present review, we describe the results of *in vitro*, animal and human investigations about the impact of miR-1290 in the development of malignancies.

## Introduction

MicroRNAs (miRNAs) are a group of small-sized transcripts with a wide range of regulatory roles. They are mostly produced through a multistep mechanism. These steps include transcription from DNA sequences into primary miRNAs and processing into precursor miRNAs and subsequently into mature miRNAs. The majority of bind with the 3′ untranslated region (3′ UTR) of target transcripts to either degrade mRNA or repress its translation. In some circumstances, miRNAs can induce translation or control transcription (O'Brien et al., 2018). Approximately 50% of all miRNAs are transcribed from intragenic regions. These miRNAs are mainly produced from introns and a number of exons of protein coding genes. Other miRNAs are intergenic and are produced in an independent manner from a host gene. Thus, these miRNAs have their own promoters (Kim and Kim, 2007; De Rie et al., 2017). miRNAs partake in the regulation of important biological functions, such as cell proliferation, differentiation and apoptosis, thus being involved in the pathoetiology of several disorders, particularly neoplastic disorders (Peng and Croce, 2016). These transcripts participate in the pathoetiology of diverse cancers (Abolghasemi et al., 2020).

miR-1290 is transcribed from *MIR1290* gene on chromosome 1p36.13. The primary transcript (NR_031622.1) has 78 nucleotides (GAG​CGU​CAC​GUU​GAC​ACU​CAA​AAA​GUU​UCA​GAU​UUU​GGA​ACA​UUU​CGG​AUU​UUG​GAU​UUU​UGG​AUC​AGG​GAU​GCU​CAA). The mature transcript of hsa-miR-1290 (MIMAT0005880) has 19 nucleotides (UGG​AUU​UUU​GGA​UCA​GGG​A). This miRNA has important functions in the carcinogenesis. Several *in vitro* studies have assessed function of miR-1290. Moreover, animal studies in lung, colon and liver cancer models have assessed functional consequences of up-regulation or silencing of this miRNA. However, some inconsistencies exist regarding the role of miR-1290. In the present manuscript, we describe the results of *in vitro*, animal and human assays about the influence of miR-1290 in the development of cancers.

## *In vitro* Studies

Forced over-expression of miR-1290 in AsPC1 and Panc5.04 pancreatic cancer cell lines has led to enhancement of cell proliferation. Inhibition of miR-1290 in pancreatic cancer cells has the reverse effects. miR-1290 mimics have also enhanced invasive properties of these cells (Li et al., 2013).

Over-expression of miR-1290 has enhanced proliferation of proliferation of lung adenocarcinoma cells and induced cell cycle progression and invasiveness. Moreover, this miRNA has suppressed cell apoptosis in this cell line. miR-1290 has been found to downregulate expression of SOCS4 to activate JAK/STAT3 and PI3K/AKT pathways (Xiao et al., 2018).

**FIGURE 1:**
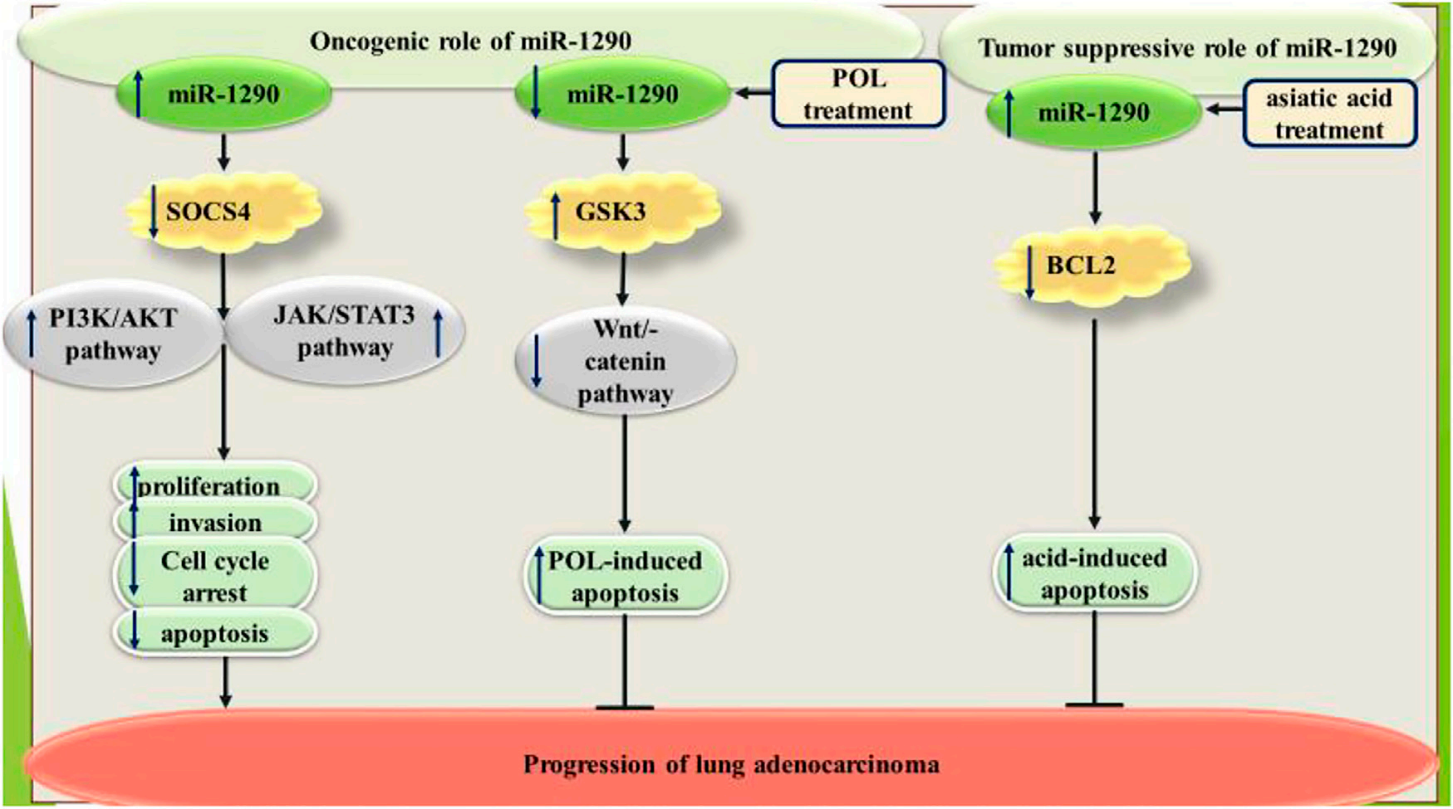
Dual roles of miR-1290 in the pathoetiology of lung cancer.

The anti-proliferative and apoptosis-inducing agent polygonatum odoratum lectin (POL) has been shown to decrease miR-1290 levels in A549 lung adenocarcinoma cells. Down-regulation of miR-1290 has been shown to increase POL-associated apoptosis in these cells. GSK3β has been found as the direct target of miR-1290 in A549 cells (Wu et al., 2016). Conversely, miR-1290 has been shown to sensitize A549 cells to the apoptosis-inducing agent asiatic acid through negatively regulating expression of BCL2. Expression of miR-1290 has been up-regulated by asiatic acid. Most notably, the apoptosis-inducing effect of asiatic acid relies on miR-1290 activity. Taken together, miR-1290 has been shown to suppress viability and cell cycle progression of A549 cells (Kim et al., 2014). Figure 1 summarizes the effects of miR-1290 in the pathoetiology of lung cancer.

In oral squamous cell carcinoma, miR-1290 has been shown to be up-regulated parallel with downregulation of CCNG2. miR-1290 silencing has inhibited metastatic ability and epithelial-mesenchymal transition (EMT). CCNG2 has been identified as the direct target of miR-1290 (Qin et al., 2019). Functional studies in laryngeal squamous cell carcinoma has shown that miR-1290 targets two tumor suppressor genes, namely ITPR2 and MAF (Janiszewska et al., 2015).

**FIGURE 2:**
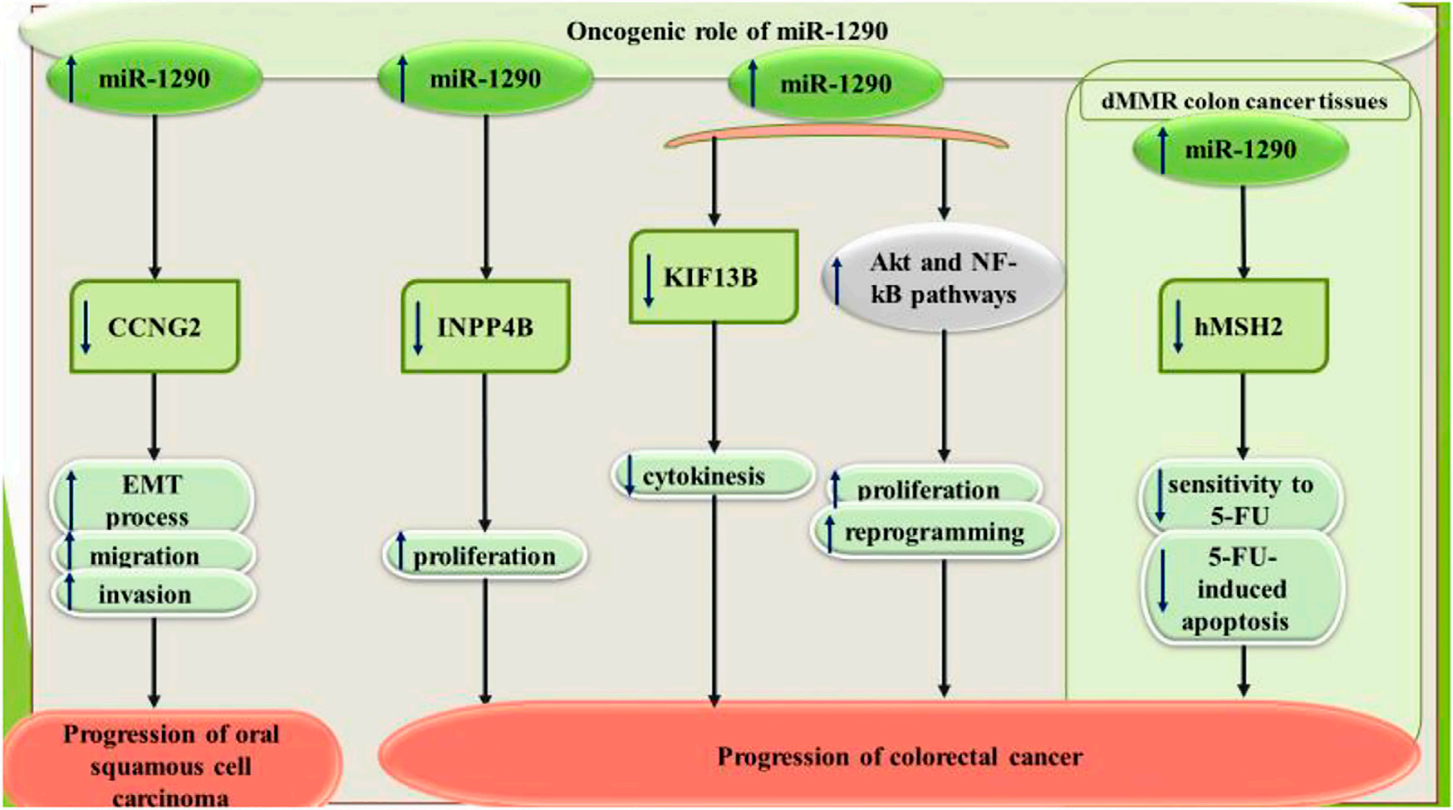
Oncogenic influence of miR-1290 in squamous cell carcinoma and colorectal cancer.

miR-1290 has oncogenic roles in colorectal cancer. miR-1290 silencing has suppressed proliferation of colorectal cancer cells. miR-1290 up-regulation has decreased expression of p27 and enhanced transcript and protein amounts of cyclin D1. NPP4B has been recognized as the target of miR-1290 (Ma et al., 2018). Moreover, miR-1290 silencing has improved cytotoxic effects of 5-fluouracil in colorectal cancer cells through targeting hMSH2 (Ye et al., 2017). Figure 2 shows the oncogenic role of miR-1290 in squamous cell carcinoma and colorectal cancer.

Exosomal miR-1290 has been found to be high in gastric cancer cell lines. miR-1290-containing exosomes could promote proliferation, migratory aptitude, and invasiveness of gastric cancer cells. NKD1 has been identified as the direct target of miR-1290 in these cells (Huang et al., 2019). Moreover, miR-1290 has been revealed to increase proliferation and migratory aptitude of gastric cancer cells through targeting FOXA1 (Lin et al., 2016).

miR-1290 has also been shown to be overexpressed in B-acute lymphoblastic leukemia (ALL) cell line SUP-B15. The anticancer agent resveratrol has been found to down-regulate expression of miR-1290 and enhance IGFBP3 levels in the ALL cells. miR-1290 can target 3′ UTR of IGFBP3 (Zhou et al., 2017). Besides, exosomal miR-1290 has been demonstrated to promote angiogenic processes in hepatocellular carcinoma through influencing expression of SMEK1 (Wang et al., 2021c).

On the other hand, miR-1290 has been shown to exert tumor suppressive role in ovarian cancer. In fact, the oncogenic long non-coding RNA (lncRNA) CCAT1 facilitates ovarian carcinogenesis through decreasing miR-1290 levels (Lai and Cheng, 2018).

**FIGURE 3:**
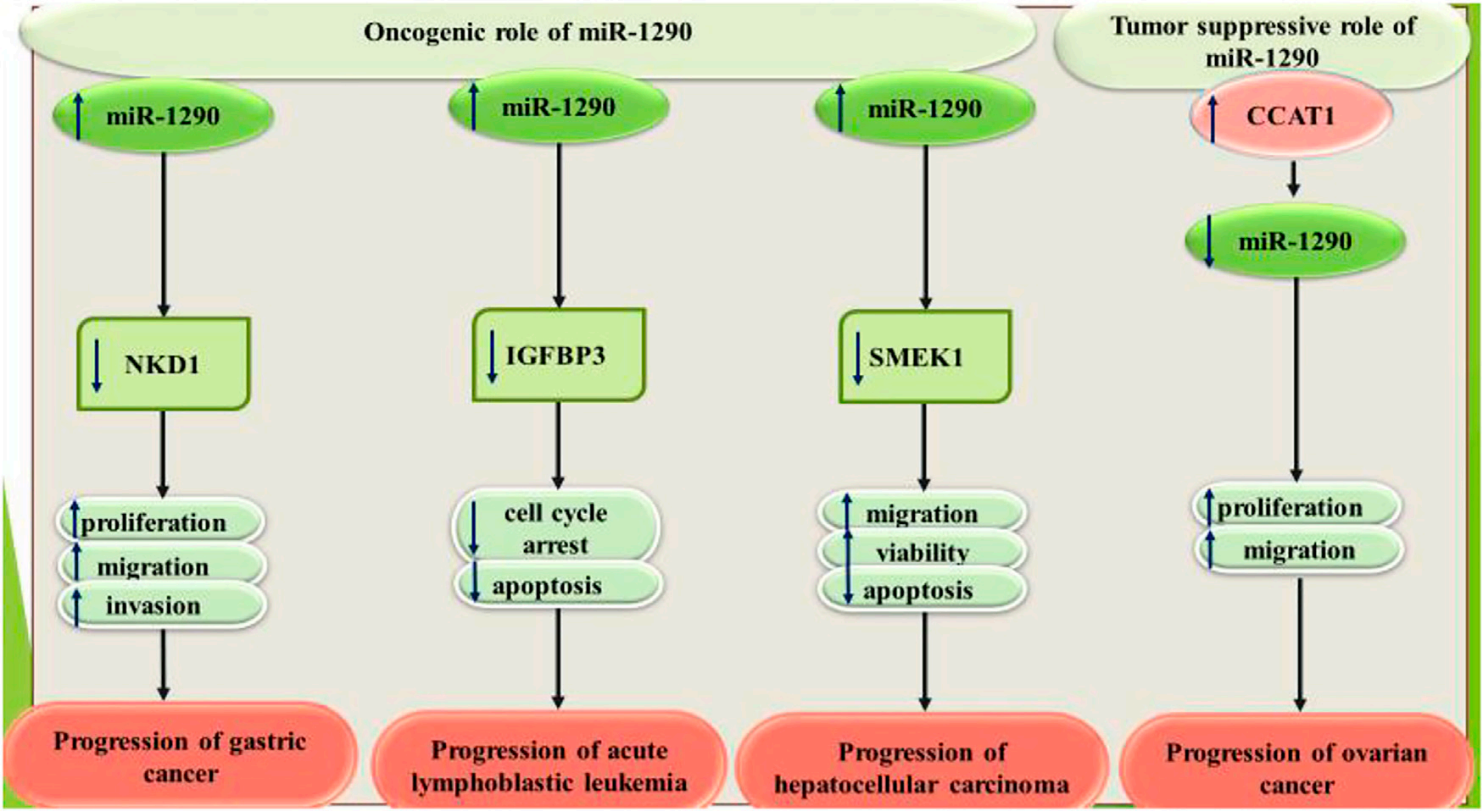
miR-1290 has oncogenic roles in gastric cancer, acute lymphoblastic leukemia and hepatocellular carcinoma, while it has tumor suppressive roles in ovarian cancer.

Figure 3 shows the roles of miR-1290 in gastric cancer, ALL, hepatocellular carcinoma and ovarian cancer.

**FIGURE 4:**
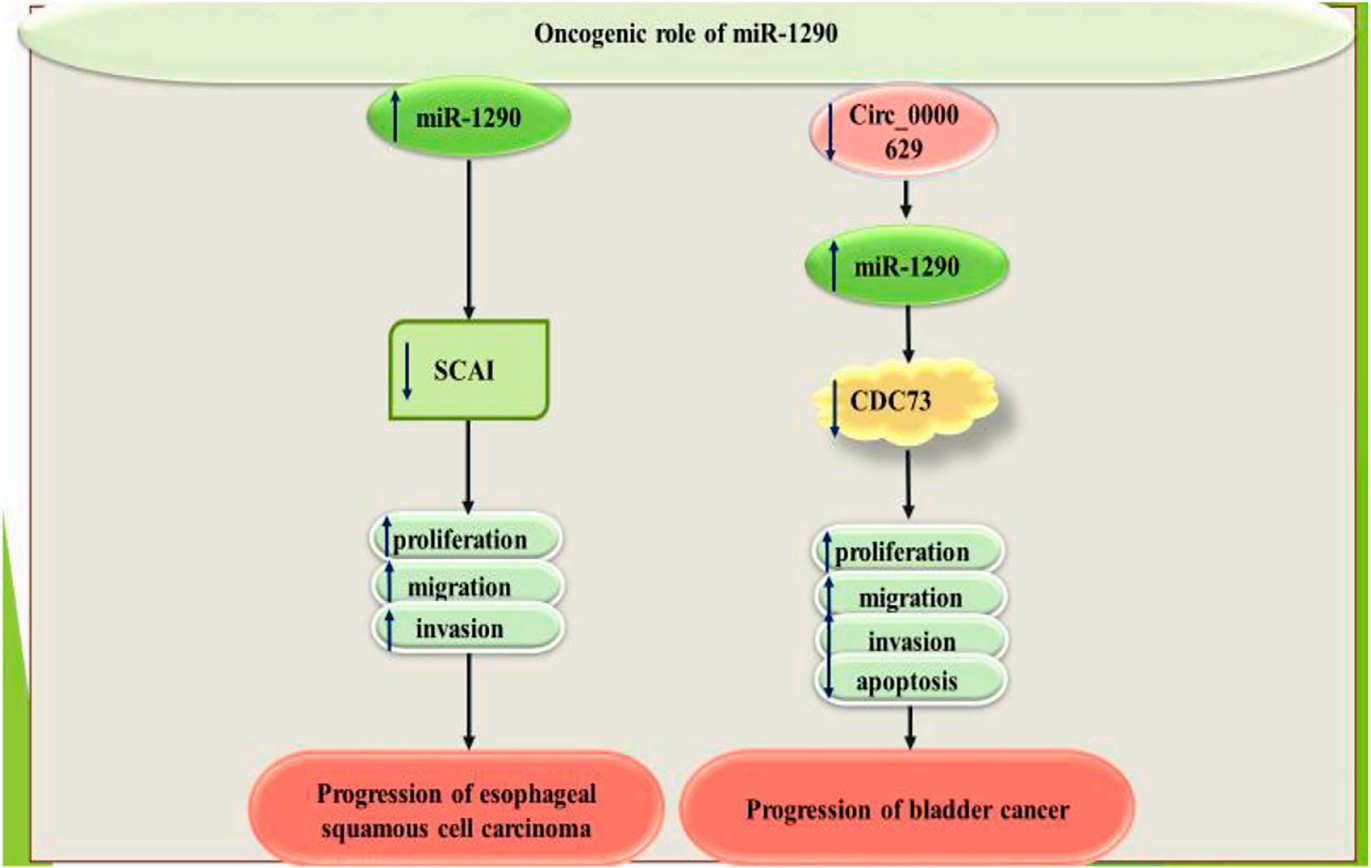
Oncogenic role of miR-1290 in esophageal and bladder cancers.

Over-expression of miR-1290 has enhanced esophageal squamous cell carcinoma growth, migration and invasiveness through decreasing SCAI levels (Li et al., 2015). In bladder cancer cells, tumor suppressor Circular RNA circ_0000629 has been shown to exert its effects through suppressing miR-1290 levels and up-regulating CDC73 expression (Wang et al., 2021b). Figure 4 shows the oncogenic role of miR-1290 in esophageal and bladder cancers.

Summary of *in vitro* studies regarding the role of miR-1290 in the carcinogenesis is provided in Table 1.

**TABLE 1:** Expression pattern of miR-1290 in cancer cell lines (∆: knock-down or deletion, POL: Polygonatum odoratum lectin, 5-FU: 5-Fluorouracil).

Tumor type	Targets/Regulators and signaling pathways	Cell line	Function	References
Pancreatic cancer	—	Panc5.04, Panc8.13, Panc10.05, Panc198, HPDE	↑ miR-1290: ↑ proliferation, ↑ invasion	Li et al. (2013)
Lung cancer	SOCS4, JAK/STAT3 signaling pathway, PI3K/AKT signaling pathway	BEAS-2B, A549, SPC-A1	↑ miR-1290: ↑ proliferation, ↑ invasion, ↓ G1/G0 phase arrest, ↓ apoptosis	Xiao et al. (2018)
	GSK3, Wnt/-catenin pathway	A549	POL treatment: ↓ miR-1290	Wu et al. (2016)
			↑ miR-1290 + POL treatment: ↓ POL-induced apoptosis	
			∆ miR-1290 + POL treatment: ↑ POL-induced apoptosis	
			↑ miR-1290: did not affect proliferation, did not affect autophagy	
			∆ miR-1290: did not affect proliferation, did not affect autophagy	
	BCL2	A549	asiatic acid treatment: ↑ miR-1290	Kim et al. (2014)
			↑ miR-1290: ↑ acid-induced apoptosis	
Oral squamous cell carcinoma	CCNG2	NHOK, Cal-27, SCC-9, SCC-25, Tca-8113 c	∆ miR-1290: ↓ migration, ↓ invasion	Qin et al. (2019)
			↑ miR-1290: ↑ EMT process	
Laryngeal squamous cell carcinoma	KIF13B	UT-SCC-34	—	Janiszewska et al. (2015)
Colorectal cancer	INPP4B	FHC, and CRC cells SW480, HT-29, COLO205, SW403, KM202L, SW620	↑ miR-1290: ↑ proliferation	Ma et al. (2018)
			∆ miR-1290: ↓ proliferation	
	—	Caco2, DLD1, HT29, LoVo, SW480	∆ miR-1290: ↓ proliferation, ↓ migration, ↓ invasion	Imaoka et al. (2016)
	hMSH2	RKO, SW480, HCT116, and LoVo	↑ miR-1290: ↑ viability, ↓ sensitivity to 5-FU	Ye et al. (2017)
			∆ miR-1290: ↑ sensitivity to 5-FU, ↑ apoptosis	
	KIF13B, Akt and NF-kB pathways	SW620, 293T, SGC7901 c	↑ miR-1290: ↑ proliferation, ↑ reprogramming, ↓ cytokinesis	Wu et al. (2013)
Gastric cancer	NKD1	SGC7901, AGS, and BGC823, GES	↑ miR-1290: ↑ proliferation, ↑ invasion, ↑ migration	Huang et al. (2019)
	FOXA1	GES-1, SGC-7901	∆ miR-1290: ↓ proliferation, ↓ migration, no significant difference in apoptosis	Lin et al. (2016)
Acute lymphoblastic leukemia	IGFBP3	PBMCs	∆ miR-1290: ↑ cell cycle arrest, ↑ apoptosis	Zhou et al. (2017)
Ovarian cancer	CCAT1	OVCAR-8, SKOV-3 w, IOSE386, OMC685	∆ lncRNA CCAT1 (which sponges miR-1290): ↓ proliferation, ↓ migration	Lai and Cheng, (2018)
Breast cancer	FOXA1, NAT1	T47D, MCF-7	↑ miR-1290: ↓ expression levels of FOXA1 and NAT1 in ER-positive breast cancer cells	Endo et al. (2013)
Hepatocellular carcinoma	SMEK1	HUVECs, Hep3 B, HepG2, SMMC-7721, PLC/PRF/5, L-02	↑ miR-1290: ↑ migration, ↑ viability, ↑ capacity of HUVECs to form tube-like structures	Wang et al. (2021c)
			∆ miR-1290: ↓ migration, ↓ viability, ↑ apoptosis	
Esophageal squamous cell carcinoma	SCAI	Eca109, TE13	↑ miR-1290: ↑ proliferation, ↑ invasion, ↑ migration	Li et al. (2015)
Chordoma	NONHSAT024778, Robo1	U-CH1	↑ NONHSAT024778 (which sponges miR-1290): ↑ proliferation, ↑ invasion, ↑ migration	Wang et al. (2021a)
Nasopharyngeal carcinoma	ZNF667-AS1, ABLIM1	NP69, c666-1, CNE-1, CNE-2, HNE1	↑ miR-1290: ↑ proliferation, ↑ invasion, ↑ migration, ↓ apoptosis	Chen et al. (2020)
Bladder cancer	Circ_0000629, CDC73	T24, SW780	↑ miR-1290: ↑ growth, ↑ invasion, ↑ migration, ↓ apoptosis	Wang et al. (2021b)

Studies in animal models.

miRNA-1290 has important roles in determination of response of cancer cells to 5-fluouracil. miR-1290 silencing has improved cytotoxic effects of 5-fluouracil in xenografts models of this cancer via targeting hMSH2 (Ye et al., 2017). Other studies have shown oncogenic roles of miR-1290 in animal models of lung cancer (Xiao et al., 2018), hepatocellular carcinoma (Wang et al., 2021c) and nasopharyngeal carcinoma (Chen et al., 2020) (Table 2). On the other hand, animal studies have shown that the oncogenic lncRNA NONHSAT024778 acts through sponging miR-1290, thus revealing a tumor suppressor role for miR-1290 (Wang et al., 2021a).

**TABLE 2:** Impact of miR-1290 in carcinogenesis based on investigations in animal models (∆: knock-down or deletion).

Tumor type	Animal models	Results	References
Lung cancer	BALB/c-nu/nu nude mice	↑ miR-1290: ↑ tumor volume, ↑ tumor weight, ↑ invasion, ↑ metastasis	Xiao et al. (2018)
Colon cancer	male BALB/c nude mice	∆ miR-1290: ↑ 5-FU-induced apoptosis	Ye et al. (2017)
Hepatocellular carcinoma	male BALB/c and NOD-SCID mice	∆ miR-1290: ↓ tumor volumes, ↓ tumor weights, ↓ proliferation, ↑ apoptosis	Wang et al. (2021c)
Chordoma	male Balb/c NOD nude mice	∆ NONHSAT024778 (which sponges miR-1290): ↓ tumor volumes, ↓ tumor weights, ↓ tumor growth	Wang et al. (2021a)
Nasopharyngeal carcinoma	BALB/c nude mice	∆ miR-1290: ↓ tumor volumes, ↓ tumor weights	Chen et al. (2020)

## Studies in Clinical Samples

In lung adenocarcinoma tissues, expression of miR-1290 has been negatively correlated with SOCS4 levels. Expression of SOCS4 has been inversely correlated with higher clinical stages and lymph node metastases (Xiao et al., 2018). Moreover, miR-1290 levels have been associated with clinicopathological landscapes and poor prognosis of patients with oral squamous cell carcinoma (Qin et al., 2019). In laryngeal squamous cell carcinoma, a high throughput miRNA profiling experiment has shown up-regulation of 33 miRNAs, among them being miR-1290 (Janiszewska et al., 2015).

Comparison of miRNA profiles between deficient and proficient mismatch repair colon cancer tissues has shown up-regulation of miR-1290 in deficient mismatch repair colorectal cancer tissues. Expression of miR-1290 has been correlated with poor prognoses of colon cancer in stages II and III patients who took 5-fluouracil-based chemotherapeutics regimens (Ye et al., 2017).

miR-1290 has also been exhibited to be up-regulated in serum exosomes of gastric cancer patients compared with healthy people (Huang et al., 2019). Another study in gastric cancer patients has shown correlation between miR-1290 over-expression and clinical stage, deepness of invasion and lymph node positivity (Lin et al., 2016).

miR-1290 has also been shown to be upregulated in esophageal squamous cell carcinoma tissues compared with unaffected neighboring samples. Over-expression of miR-1290 has been associated with level of differentiation, N classification TNM stage in this type of esophageal cancer (Li et al., 2015).

On the other hand, in oral squamous cell carcinoma, levels of this miRNA has been reported to be decreased in blood samples of patients compared with control samples (Nakashima et al., 2019). Moreover, expression of miR-1290 has been reported to be decreased in chordoma samples (Wang et al., 2021a). Table 3 summarizes the results of studies that reported dysregulation of miR-1290 in clinical samples.

**TABLE 3:** Dysregulation of miR-1290 in clinical specimens (DC: benign pancreatic disease controls, PFS: progression free survival, LUAD: Lung adenocarcinoma, ANCTs: adjacent non-cancerous tissues, OS: Overall survival, DFS: disease-free survival, TNM: tumor-node-metastasis, NSCLC: non-small-cell lung cancer, CRA: colorectal adenoma, HGSOC: high grade serous ovarian cancer, EOC: epithelial ovarian cancer, HGSOC: high grade serous ovarian carcinoma.).

Tumor type	Samples	Expression (tumor vs. Normal)	Kaplan-Meier analysis (impact of miR-1290 up-regulation)	Univariate/Multivariate cox regression	Association of miR-1290 expression with clinicopathologic characteristics	References
Prostate cancer	23 CRPC patients	up	Poor OS	—	—	Huang et al. (2015)
Pancreatic cancer (PC)	GEO datasets: (GSE113486 and GSE106817)	up	—	—	—	Wei et al. (2020)
	120 PC patients, 40 DC patients, and 40 healthy controls	up	—	miR-1290 expression was independent risk factors for PC.	gender (male), and stage III and IV	
	167 PC patients and 267 healthy subjects	up	shorter OS and DFS	miR-1290 was not found to be an independent negative prognostic factor for OS and DFS in PC patients	PC aggressiveness	Tavano et al. (2018)
	81 PDAC patients, 28 PNETs patients, 20 IPMN patients, 45 chronic pancreatitis patients, and 39 healthy controls	higher in patients with IPMNs than healthy controls, higher in patients with invasive pancreatic cancer than patients with IPMNs, higher in intermediate- and high-grade dysplasia than those with low-grade dysplasia	—	—	—	Li et al. (2013)
Lung cancer	70 LUAD patients and 40 healthy controls	up	shorter PFS	The level of miR-1290 was an independent prognostic factor in LUAD patients	gender (male), advanced TNM stage, tumor size, lymph node metastasis, distant metastasis, smoking, and drinking	Wu et al. (2020)
	32 pairs of LUAD tissues and ANCTs	up	—	—	—	Xiao et al. (2018)
	33 pairs of NSCLC tissues and ANCTs	up	shorter OS	—	stage IIIa, lymph node metastasis	Mo et al. (2015)
	serum samples from 73 NSCLC patients, 19 patients with various benign lung disease, 34 healthy controls	up	shorter OS	TNM stage and lymph node metastasis status and serum miR-1290 expression were found to be the independent prognostic factors for OS.	TNM stage, lymph node metastasis	
Oral squamous cell carcinoma (OSCC)	47 pairs of OSCC tissues and ANCTs	up	shorter OS	—	TNM stage and the lymph node metastasis	Qin et al. (2019)
	10 OSCC patients and 10 healthy volunteers	down	—	—	—	Nakashima et al. (2019)
	plasma samples from 55 OSCC patients	down	higher OS and DFS	Expression OF miR-1290 was found to be a significant prognostic factor for OSCC patients	tumor differentiation and response to CRT	
Laryngeal squamous cell carcinoma (LSCC)	50 LSCC patients and 5 epithelial no tumor controls	up	—	—	—	Janiszewska et al. (2015)
	5 pairs of LSCC tissues and ANCTs	up	—	—	—	Sun et al. (2013)
	48 LSCC patients	up	—	—	—	
Colorectal cancer (CRC)	GEO datasets: (GSE108153, GSE81581, GSE55139 and GSE41655)	up	—	—	—	Liu et al. (2019)
	15 CRC patients, 15 adenoma cases and 15 healthy controls	up	—	—	—	
	80 CRC patients, 50 adenoma cases, and 30 healthy controls	up	—	—	larger tumor size, advanced TNM stage, lymph node metastasis, and distant metastasis	
	8 pairs of CRC tissues and ANCTs	up	—	—	—	Ma et al. (2018)
	20 normal colon samples and 50 CRC samples	up	—	—	—	
	12 pairs of CRC tissues and ANCTs, and 12 colorectal adenomas tissues	up	poorer OS	High miR-1290 expression, large tumor size, lymphatic invasion, venous invasion, high T stage, lymph node metastasis, distant metastasis, and high carcinoembryonic antigen levels were associated with poor OS.	—	Imaoka et al. (2016)
	serum samples from 12 CRC patients,12 adenoma patients, and 12 healthy persons	up	worse OS	Increased serum miR-1290 level, poor differentiation, lymphatic invasion, venous invasion, high T stage, lymph node metastasis, distant metastasis, and high CEA levels were associated with poor OS.	—	
	serum samples from 211 CRC patients, 56 colorectal adenoma patients, and 57 healthy controls	up	—	—	stage IV, tumor size, serosal invasion, lymphatic and venous invasion, and metastasis	
	GEO database: GSE39833 (88 CRC patients and 11 healthy controls)	up	—	—	—	Li et al. (2016)
Colorectal cancer (CRC)	54 CRA patients	up	—	—	adenoma size	Handa et al. (2021)
Colon cancer	291 colon cancer tumor tissues	up	Lower OS and DFS	miR-1290 expression, N stage, AJCC stage, tumor differentiation, vascular invasion, miR-and MMR status were associated with decreased OS and DFS.	dMMR Status, tumor location, N stage, and tumor differentiation	Ye et al. (2017)
	25 pairs of colon cancer tissues and ANCTs	up	—	—	—	Wu et al. (2013)
Gastric cancer (GC)	serum samples from 20 GC patients and 10 healthy controls	up	—	—	—	Huang et al. (2019)
	20 pairs of GC tissues and ANCTs	up	—	—	advanced clinical staging and depth of tumor invasion	Lin et al. (2016)
Acute lymphoblastic leukemia (ALL)	15 ALL patients and 15 healthy controls	IGFBP3 (a target of miR-1290) expression is decreased	—	—	—	Zhou et al. (2017)
Ovarian cancer (OC)	sera samples from 70 EOC patients and 13 healthy controls	no significant difference	—	—	—	Kobayashi et al. (2018)
	30 HGSOC patients and 13 healthy controls	up	—	—	tumor burden	
	40 pairs of OC tissues and ANCTs	upregulation of lncRNA CCAT1 (which sponges miR-1290)	higher CCAT1 = shorter OS	—	tumor size and lymph node metastasis	Lai and Cheng, (2018)
Breast cancer	blood samples from 60 breast cancer patients and 20 healthy controls	up	—	—	lymph node metastasis and Stage II/III	Li et al. (2021)
	4 ER-high Ki67-low tumor tissues and 4 ER-low Ki67-high tumor tissues	down in ER-high Ki67-low tumors	—	—	tumor grade	Endo et al. (2013)
Hepatocellular carcinoma (HCC)	49 pairs of HCC tissues and ANCTs	Up	—	—	—	Wang et al. (2021c)
	serum samples of 49 HCC patients and serum samples of 28 healthy controls	Up	—	—	—	
Esophageal squamous cell carcinoma (ESCC)	24 pairs of ESCC tumor tissues and ANCTs	up	—	—	differentiation, N classification and tumor-node-metastasis stage	Li et al. (2015)
Chordoma	20 chordoma tissues and 10 FNP tissues	down	—	—	—	Wang et al. (2021a)
Nasopharyngeal carcinoma (NPC)	GEO database: (GSE70970)	up	—	—	—	Chen et al. (2020)
Cutaneous squamous cell carcinoma (cSCC)	8 cSCC patients and 8 controls	up	—	—	—	Geusau et al. (2020)
Cervical cancer	sera from 6 cervical cancer patients and 6 healthy persons	up	—	—	—	Nagamitsu et al. (2016)
	Sera of 20 cervical cancer patients 10 healthy persons	up	—	—	—	
	serum samples from 100 cervical cancer patients and 31 healthy controls	up	—	—	—	
	microarray analysis	up in cells with HPV infection upon 5-AZA treatment	—	—	—	Yao et al. (2013)

Serum levels of miR-1290 have been shown to be higher in patients with intraductal papillary mucinous pancreatic cancer compared with healthy subjects. The ability of serum levels of miR-1290 in separation of patients with low-stage pancreatic cancer from controls has been higher than CA19-9. Notably, higher levels of miR-1290 has been predictive of poor outcome following pancreaticoduodenectomy (Li et al., 2013). In this type of cancer, miR-1290 has been shown to appropriately distinguish neoplastic condition from both healthy condition and chronic pancreatitis (Wei et al., 2020). In colorectal cancer, levels of this miRNA could distinguish cancer status from healthy condition with up to ideal diagnostic power. Moreover, it can separate colorectal adenoma from healthy status with lower values (Imaoka et al., 2016). Table 4 shows the diagnostic value of miR-1290 in cancers.

**TABLE 4:** Diagnostic value of miR-1290 in cancers (PC: pancreatic cancer; DC: benign pancreatic disease control; LUAD: Lung adenocarcinoma, EOC: epithelial ovarian cancer, HGSOC: high grade serous ovarian carcinoma).

Tumor type	Samples	Distinguish between	Area under curve	Sensitivity (%)	Specificity (%)	References
Pancreatic cancer (PC)	120 PC patients and 40 healthy controls	PC patients vs. healthy controls	0.93	75.0	97.5	Wei et al. (2020)
	120 PC patients and 40 DC	PC patients vs. DC	0.89	88.3	72.5	
	120 PC patients and controls	PC patients vs. all controls	0.91	74.2	91.2	
	81 PDAC patients and 39 healthy controls	PDAC patients vs. healthy controls	0.96	—	—	Li et al. (2013)
	81 PDAC patients and 45 chronic pancreatitis samples	PDAC patients vs. chronic pancreatitis samples	0.81	—	—	
	81 PDAC patients and 28 PNETs patients	PDAC patients vs. PNET samples	0.80	—	—	
	81 PDAC patients and all controlls	PDAC patients vs. all controls	0.85	—	—	
Lung cancer	70 LUAD patients and 40 healthy controls	LUAD patients vs. controls	0.937	80.0	96.7	Wu et al. (2020)
Colorectal cancer (CRC)	15 CRC patients, 15 colorectal adenoma patients and 15 healthy controls	CRC patients vs. healthy controls	0.96	78.79	93.33	Liu et al. (2019)
		colorectal adenoma patients vs. healthy controls	0.92	79.66	86.67	
	12 CRC patients,12 colorectal adenoma patients, and 12 healthy controls	CRC patients vs. healthy controls	1.000	100	100	Imaoka et al. (2016)
		colorectal adenoma patients vs healthy controls	0.722	50	100	
	211 CRC patients, 56 colorectal adenoma patients, and 57 healthy controls	CRC patients vs. healthy controls	0.830	70.1%	91.2	
		colorectal adenoma patients vs. healthy controls	0.718	46.4	91.2	
Ovarian cancer (OC)	sera samples from 70 EOC patients and 13 healthy controls	EOC patients vs. healthy controls	0.48	0.51	0.57	Kobayashi et al. (2018)
	30 HGSOC patients and 13 healthy controls	HGSOC patients vs. healthy controls	0.71	0.63	0.85	

## Discussion

Several miRNAs have been found to influence the carcinogenesis. miR-1290 is an example of oncomiRs based on the bulk of relevant evidence. This miRNA has interactions with several cancer-related mRNAs such as SOCS4, GSK3, BCL2, CCNG2, KIF13B, INPP4B, hMSH2, KIF13B, NKD1, FOXA1, IGFBP3, FOXA1, NAT1, SMEK1, SCAI, ZNF667-AS1, ABLIM1, and CDC73.

Moreover, miR-1290 has interactions with a number of non-coding RNAs such as Circ_0000629, CCTA1 and NONHSAT024778. The interaction between lncRNAs/circRNAs and miRNAs has important implications in pathoetiology of cancers, thus future studies are needed to identify other non-coding RNAs that interact with miR-1290 in the context of neoplastic conditions. In fact, these lncRNAs and circRNAs can act as sponge for miRNAs to decrease its bioavalability, thus enhancing expression of targets of miR-1290. Therefore, they construct a competing endogenous RNA (ceRNA) network.

In addition to its role in the regulation of gene expression, miR-1290 can regulate activity of JAK/STAT3, PI3K/AKT, Wnt/β-catenin and NF-κB signaling pathways, thus influencing several cancer-related routes.

Most evidence indicates the oncogenic roles of miR-1290, yet controversial evidence also exists. Particularly, in the lung cancer, both oncogenic and tumor suppressor roles have been reported for miR-1290.

A number of anticancer agents such as POL, asiatic acid and resveratrol has been shown to affect expression of miR-1290. Moreover, this miRNA can influence response of neoplastic cells to the chemotherapeutic agent 5-fluouracil. Thus, one can deduce that miR-1290-targeting strategies can modulate response of cancer cells to a wide variety of antineoplastic modalities.

In addition to its therapeutic implications, the existence of miR-1290 in cancer-derived exosomes not only indicates its application in diagnostic approaches, but also shows the effect of these vehicles in conferring neoplastic features inside the tumor bulk.

The ceRNA networks constructed by circRNAs, miR-1290 and target mRNAs can be used as prognostic biomarkers and therapeutic targets in different cancers. These ceRNA networks are superior to single transcripts since they reflect a more comprehensive overview of dysregulated pathways. Theoretically, the ceRNA regulatory networks including lncRNAs or circRNAs-miR-1290-mRNAs can be applied as prognostic biomarkers and therapeutic targets in different cancers. High throughput sequencing methods have facilitated applicability of these networks in diagnostic, prognostic and therapeutic fields. Moreover, these techniques have facilitated design of personalized therapeutic options based on the identified dysregulated networks in samples obtained from each patient. Application of this data can enhance survival of patients.

Cumulatively, miR-1290 is a cancer-related miRNA with possible application as diagnostic and prognostic marker in diverse types of cancers. Therapeutic applications of anti-miR-1290 modalities should be assessed in future. Moreover, future studies should address the possibility of targeting the miR1290-containg ceRNA networks.
